# A Preliminary Investigation into the Effects of Immunosuppressive Therapy on Outcomes of Endovascular Aneurysm Repair

**DOI:** 10.3400/avd.oa.25-00134

**Published:** 2026-04-18

**Authors:** Yohei Yamamoto, Toru Kikuchi, Kazuki Tsukuda, Natsuho Maekawa, Ai Kazama, Yoshiki Wada, Tsuyoshi Ichinose, Toshifumi Kudo

**Affiliations:** Division of Vascular Surgery, Department of Cardiovascular Surgery, Institute of Science Tokyo, Tokyo, Japan

**Keywords:** abdominal aortic aneurysm, endovascular aneurysm repair, immunosuppressive therapy, postoperative complication

## Abstract

**Objectives:**

This study aimed to evaluate the short- and long-term outcomes of endovascular aneurysm repair (EVAR) for abdominal aortic aneurysms (AAAs), with a particular focus on the impact of immunosuppressive therapy.

**Methods:**

The authors retrospectively reviewed the institutional data of patients with AAAs who underwent EVAR between 2008 and 2023.

**Results:**

A total of 307 consecutive patients underwent elective EVAR for AAA. Of these, 26 patients (8.5%) were receiving immunosuppressive therapy (IT group). Patients in the IT group had a significantly higher incidence of early postoperative complications than those in the non-IT group (42.3% vs. 17.4%). The mean follow-up period was 40.7 ± 33.6 months. The cumulative incidence of sac shrinkage was similar between the groups, whereas the cumulative incidence of sac expansion was significantly higher in the IT group. The rates of reintervention and open aneurysmorrhaphy were also significantly higher in the IT group. The use of immunosuppressive therapy was one of the significant risk factors for late sac expansion (hazard ratio, 4.66, 95% confidence interval, 2.12–10.23). Overall survival was similar regardless of immunosuppressive therapy status.

**Conclusions:**

Immunosuppressive therapy may be associated with an increased risk of early postoperative complications and late sac expansion in patients undergoing EVAR.

## Introduction

Endovascular aneurysm repair (EVAR) is typically indicated for patients who are poor candidates for open surgical repair of an abdominal aortic aneurysm (AAA). Immunosuppressive therapy is one of the factors that may lead to the selection of EVAR over open repair. Immunosuppressive agents, including corticosteroids, suppress the immune system and increase tissue fragility,^1,2)^ and have been associated with a higher risk of complications following invasive procedures, particularly wound-related and infectious complications. In addition, immunosuppressive agents are thought to influence the growth of AAAs, as inflammation plays a key role in AAA pathogenesis.^3)^

Therefore, immunosuppressive therapy may affect the outcomes of AAA treatment. However, its impact on postoperative outcomes in patients undergoing EVAR remains unclear.

This study aimed to evaluate the short- and long-term outcomes of EVAR for AAA, with a particular focus on the impact of immunosuppressive therapy.

## Materials and Methods

This was a single-center, retrospective study. We collected clinical data on consecutive patients who underwent elective EVAR for AAA at Tokyo Medical and Dental University Hospital (currently Institute of Science Tokyo Hospital) between January 2008 and December 2023. Emergency cases and radiologically suspected inflammatory aneurysms were excluded. Clinical data, including patient demographics, comorbidities, intraoperative parameters, and clinical outcomes, were retrospectively collected from electronic medical records and subsequently analyzed. This study was conducted in accordance with the ethical principles of the Declaration of Helsinki. The ethics committee of the Institute of Science Tokyo Hospital approved this study (Reference number: M2021-279).

EVAR was, in principle, applied to patients aged ≥75 years with a high degree of surgical risk, unless the patient had unsuitable anatomy. Immunosuppressive therapy was defined as chronic use of corticosteroids, immunosuppressive or immunomodulatory agents, or a combination of these, administered for at least 6 months prior to EVAR. Patients were divided into 2 groups: those receiving immunosuppressive therapy (IT group) and those not receiving such therapy (non-IT group). Clinical outcomes were then compared between the 2 groups.

Early postoperative complications were defined as any morbidities occurring within 30 days after EVAR.

Postoperative imaging follow-up using computed tomography or duplex ultrasound was conducted at 1 week, 3 months, 6 months, and 1 year postoperatively, and annually thereafter. Postoperative sac shrinkage and expansion were defined as a decrease or increase of ≥5 mm in the sac diameter compared to the preoperative diameter, respectively.

During follow-up, type I and III endoleaks were promptly treated with appropriate methods, such as additional graft placement, whenever they were identified. Persistent or late-onset type II endoleaks and associated sac expansion were initially managed with endovascular treatment whenever feasible. Open aneurysmorrhaphy was considered when there was an increase in sac diameter of ≥10 mm or a rapid sac expansion of ≥5 mm per year, following unsuccessful endovascular treatment. Primary outcomes of the present study included early postoperative outcomes, aneurysm sac behavior during follow-up, and long-term survival.

### Statistical analyses

Continuous variables were expressed as the mean value ± standard deviation. The clinical characteristics of the 2 groups were compared using the χ^2^ test or Fisher’s exact test for categorical data and the Mann–Whitney U test for continuous data. The incidence of sac shrinkage and sac expansion, the incidence of reintervention and open aneurysmorrhaphy, and overall survival rates were analyzed using the Kaplan–Meier method. A Cox regression analysis was used to identify risk factors for late sac expansion. All statistical analyses were performed using BellCurve for Excel (Social Survey Research Information, Tokyo, Japan).

## Results

During the study period, a total of 307 consecutive patients underwent elective EVAR for AAA, of whom 26 patients (8.5%) were receiving immunosuppressive therapy. The patients’ demographic factors and preoperative comorbidities are shown in **Table 1**. The prevalence of diabetes mellitus and coronary artery disease was significantly higher in the IT group. **Table 2** summarizes the clinical characteristics of patients in the IT group. The most common underlying disease requiring immunosuppressive therapy was rheumatoid arthritis, followed by interstitial pneumonia and medium- or small-vessel vasculitis. The immunosuppressive regimens consisted of corticosteroids alone in 16 patients, an immunomodulatory agent alone in 1 patient, and a combination of corticosteroids and immunosuppressive or immunomodulatory agents in 9 patients.

**Table 1:** Demographics and preoperative comorbidities of the study population

	All subjects (n = 307)	IT group (n = 26)	Non-IT group (n = 281)	p-Value
Age (years)	77.0 ± 7.9	74.3 ± 7.9	77.2 ± 7.9	0.127
Male	256 (83.4)	17 (65.4)	239 (85.1)	0.010
Hypertension	219 (71.3)	17 (65.4)	202 (71.9)	0.483
Dyslipidemia	139 (45.3)	15 (57.7)	124 (44.1)	0.184
Diabetes mellitus	58 (18.9)	10 (38.5)	48 (17.1)	0.008
Coronary artery disease	72 (23.5)	11 (42.3)	61 (21.7)	0.018
Cerebrovascular disease	54 (17.6)	4 (15.4)	50 (17.8)	1.000
COPD	54 (17.6)	8 (30.8)	46 (16.4)	0.065
Chronic kidney disease	132 (43.0)	15 (57.7)	117 (41.6)	0.114
Antiplatelet therapy	103 (33.6)	9 (34.6)	94 (33.5)	0.904
Anticoagulant therapy	34 (11.1)	3 (11.5)	31 (11.0)	1.000
Statin	123 (40.1)	13 (50.0)	110 (39.1)	0.280
Ever smoked	205 (66.8)	16 (61.5)	189 (67.3)	0.554

Data are expressed as the mean value ± standard deviation or number (percentage) of patients.

IT: immunosuppressive therapy; COPD: chronic obstructive pulmonary disease

**Table 2:** Clinical characteristics of patients in the IT group

	All subjects (n = 26)
Underlying disease	
Rheumatoid arthritis	7 (26.9)
Interstitial pneumonia	4 (15.4)
Medium- or small-vessel vasculitis	4 (15.4)
Chronic nephritis	2 (7.7)
Other autoimmune diseases (SLE, IgG4RD, Sjögren syndrome)	5 (19.2)
Others (malignancy, drug eruption, hypopituitarism)	4 (15.4)
Immunosuppressive regimen	
Corticosteroids alone	16 (61.5)
Immunosuppressive agent alone	1 (3.8)
Corticosteroid and immunosuppressive or immunomodulatory agents	9 (34.6)

Data are expressed as the number (percentage).

IT: immunosuppressive therapy; SLE: systemic lupus erythematosus; IgG4RT: immunoglobulin G4-related disease

**Table 3** summarizes aneurysm characteristics, EVAR-related procedural details, and early postoperative outcomes. There were no significant differences in anatomical characteristics or in the EVAR devices used between the groups. Prophylactic embolization of the inferior mesenteric artery or lumbar arteries is not routinely performed; however, it was carried out in 13 cases (4.2%) at the operator’s discretion. Thirteen postoperative complications occurred in 11 patients in the IT group within 30 days, including wound complications in 4 patients, acute kidney injury in 3, cerebral infarction in 2, lower limb ischemia in 1, and sepsis in 1. The early postoperative complication rate was significantly higher in the IT group than in the non-IT group (42.3% vs. 17.4%, p = 0.002). There was no 30-day postoperative mortality in the IT group.

**Table 3:** Aneurysm characteristics, EVAR-related procedural details, and early postoperative outcomes

	All subjects (n = 307)	IT group (n = 26)	Non-IT group (n = 281)	p-Value
Aneurysm characteristics				
Aneurysm diameter (mm)	49.7 ± 10.1	49.8 ± 9.6	49.7 ± 10.1	0.782
Proximal neck diameter (mm)	21.2 ± 3.3	21.8 ± 4.2	21.2 ± 3.2	0.240
Proximal neck length (mm)	35.1± 15.8	39.5 ± 19.5	34.7 ± 15.3	0.205
Neck characteristics outside of the IFU	39 (12.7)	6 (23.1)	33 (11.8)	0.097
EVAR device used				
Excluder	156 (50.8)	13 (50.0)	143 (50.9)	0.931
Powerlink or AFX	64 (20.8)	6 (23.1)	58 (20.6)	0.770
Aorfix	34 (11.1)	3 (11.5)	31 (11.0)	1.000
Endurant	29 (9.4)	2 (7.7)	27 (9.6)	1.000
Zenith	14 (4.6)	0 (0.0)	14 (5.0)	0.618
Alto	8 (2.6)	1 (3.8)	7 (2.5)	0.512
Treo	2 (0.7)	1 (3.8)	1 (0.4)	0.163
Prophylactic embolization				
IMA or lumbar	13 (4.2)	1 (3.8)	12 (4.3)	1.000
Postoperative complications within 30 days				
Any*	60 (19.5)	11 (42.3)	49 (17.4)	0.002
Acute kidney injury	18 (5.9)	3 (11.5)	15 (5.3)	0.187
Wound complication	10 (3.3)	4 (15.4)	6 (2.1)	0.006
Lower limb ischemia	7 (2.3)	1 (3.8)	6 (2.1)	0.465
Access complication	5 (1.6)	0 (0.0)	5 (1.8)	1.000
Cerebral infarction	4 (1.3)	2 (7.7)	2 (0.7)	0.037
Ischemic colitis	4 (1.3)	1 (3.8)	3 (1.1)	0.299
Aortic dissection	2 (0.7)	0 (0.0)	2 (0.7)	1.000
Iliopsoas abscess	2 (0.7)	0 (0.0)	2 (0.7)	1.000
Pneumonia	2 (0.7)	0 (0.0)	2 (0.7)	1.000
Myocardial infarction	1 (0.3)	0 (0.0)	1 (0.4)	1.000
Sepsis	1 (0.3)	1 (3.8)	0 (0.0)	0.085
Others	6 (1.9)	1 (3.8)	5 (1.8)	0.414
Postoperative mortality within 30 days	3 (1.0)	0 (0.0)	3 (1.1)	1.000

Data are expressed as the mean value ± standard deviation or number (percentage) of patients.

*The total number of complications listed exceeds this number because some patients had more than one complication.

EVAR: endovascular aneurysm repair; IT: immunosuppressive therapy; IFU: instruction for use; IMA: inferior mesenteric artery

The overall mean follow-up period was 40.7 ± 33.6 months. All patients in the IT group continued to receive immunosuppressive therapy at the time of final follow-up. At the final follow-up, persistent or late-onset type II endoleaks were observed in 11 patients (42.3%) in the IT group and in 82 patients (28.6%) in the non-IT group. Late-onset type Ia or Ib endoleaks were observed in 11 patients (3.6%), including 3 patients in the IT group and 8 in the non-IT group. Among these patients, all but 1 had concomitant persistent type II endoleaks. At final follow-up, 7 (26.9%) patients in the IT group showed sac shrinkage, while 8 (30.8%) patients showed sac expansion. Among the 8 patients who developed sac expansion in the IT group, 7 had persistent or late-onset type II endoleaks.

**Fig. 1:**
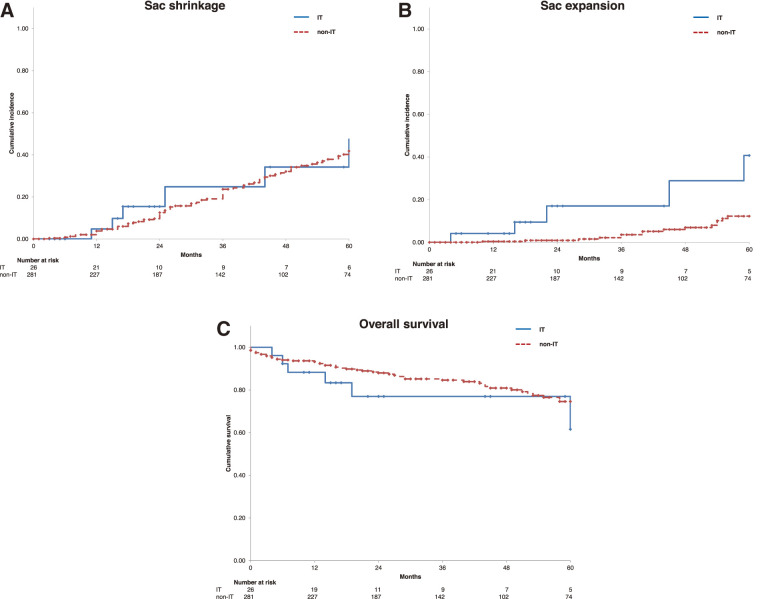
Kaplan–Meier curves showing the cumulative incidence of sac shrinkage (**A**) and sac expansion (**B**), and overall survival (**C**) in the IT and non-IT groups. IT: immunosuppressive therapy

The cumulative incidence of sac shrinkage was similar between the groups; however, the cumulative incidence rates of sac expansion were significantly higher in the IT group than in the non-IT group, at 4.2%, 17.6%, and 40.7% at 1, 3, and 5 years, respectively, compared with 0.0%, 3.6%, and 12.2% (p <0.001) (**Fig. 1A** and **1B**).

Thirty-six patients (11.7%) underwent reintervention to treat or prevent sac expansion, of whom 6 (2.0%) required open aneurysmorrhaphy. The 5-year cumulative incidence rates of reintervention and open aneurysmorrhaphy were significantly higher in the IT group, at 57.8% versus 15.7% (p <0.001) and 41.7% versus 1.8% (p <0.001), respectively.

Female sex, the use of immunosuppressive therapy, proximal neck diameter, neck characteristics outside of the instruction for use, and persistent or late-onset type II endoleaks were significant risk factors for late sac expansion (**Table 4**).

**Table 4:** Univariate analysis of risk factors for late sac expansion

Risk factor	HR (95% CI)	p-Value
Age	1.03 (0.99–1.08)	0.137
Female sex	2.83 (1.41–5.67)	0.003
Hypertension	1.87 (0.83–4.18)	0.129
Dyslipidemia	1.60 (0.88–2.91)	0.121
Diabetes mellitus	1.49 (0.73–3.03)	0.275
Coronary artery disease	1.13 (0.60–2.13)	0.695
Cerebrovascular disease	1.17 (0.61–2.23)	0.640
COPD	1.62 (0.81–3.57)	0.162
Chronic kidney disease	1.60 (0.90–2.86)	0.109
Antiplatelet therapy	0.63 (0.33–1.20)	0.163
Anticoagulant therapy	1.37 (0.58–3.26)	0.472
Statin	1.21 (0.67–2.20)	0.521
Immunosuppressive therapy	4.66 (2.12–10.23)	<0.001
Ever smoked	0.57 (0.31–1.05)	0.070
Aneurysm diameter	1.00 (0.97–1.03)	0.891
Proximal neck diameter (mm)	1.10 (1.02–1.19)	0.018
Proximal neck length (mm)	1.00 (0.98–1.02)	0.793
Neck characteristics outside of the IFU	2.57 (1.26–5.25)	0.009
Persistent or late-onset type II endoleak	5.81 (2.80–12.09)	<0.001

HR: hazard ratio; CI: confidence interval; COPD: chronic obstructive pulmonary disease; IFU: instruction for use

Nine patients (34.6%) in the IT group died during the follow-up period. The causes of death were respiratory failure in 5 patients, infection in 2, malignancy in 1, and gastrointestinal bleeding in 1. No aneurysm-related deaths were observed in this group. The overall survival rates at 1, 3, and 5 years were 88.3%, 77.0%, and 61.6% in the IT group, and 93.2%, 84.6%, and 74.6% in the non-IT group, respectively (p = 0.267) (**Fig. 1C**).

## Discussion

The present study investigated the short- and long-term outcomes of EVAR, with a particular focus on the impact of immunosuppressive therapy. The main findings were that patients receiving immunosuppressive therapy had significantly higher rates of early postoperative complications and late sac expansion.

Immunosuppressive therapy is essential for patients with specific medical conditions; however, it has several adverse effects, including susceptibility to infection, tissue fragility, delayed wound healing, and thromboembolic events.^1,2)^ These adverse effects may potentially impact clinical outcomes following invasive procedures. Several studies have examined the adverse effects of immunosuppressive therapy on surgical outcomes.

Hung et al.^4)^ conducted a meta-analysis of 17 studies to investigate the impact of chronic corticosteroid use on outcomes in orthopedic surgery and reported that it was associated with an increased risk of overall complications, especially wound and infectious complications. Similarly, Sims et al.^5)^ reported that the use of immunosuppressive therapy was associated with an increased risk of infectious complications after colectomy for colorectal cancer. While it is well recognized that immunosuppressive therapy is associated with an increased risk of postoperative adverse events in orthopedic and colorectal surgery, its association with surgical outcomes in cardiovascular disease remains unclear. Kaihara et al.^6)^ investigated the patients who underwent transcatheter aortic valve implantation for aortic valve stenosis and reported that the use of immunosuppressive agents was not associated with early postoperative complications or mid-term outcomes. In line with these findings, Pai et al.^7)^ also demonstrated that chronic corticosteroid use was not associated with increased mortality or overall morbidity following cardiac surgery.

In the present study, the rate of early postoperative complications in patients receiving immunosuppressive therapy was 42.3%, which was significantly higher than that in patients not receiving such therapy. It is possible that the adverse effects of immunosuppressive therapy, particularly corticosteroids, contributed to the significant increase in wound complications observed in patients receiving immunosuppressive therapy. Therefore, although most of these complications were not life-threatening, efforts to minimize postoperative complications are warranted. Since 2021, when the use of percutaneous closure devices (Perclose ProGlide; Abbott Vascular, Santa Clara, CA, USA) was reimbursed by the national insurance system in Japan, all patients receiving immunosuppressive therapy have undergone EVAR via a percutaneous approach, and no early postoperative complications have been observed in this subgroup. The percutaneous approach, which avoids femoral artery cut-down, may help reduce postoperative complications, particularly wound-related complications.

Another important finding of the present study is that the incidence rates of late sac expansion, reintervention, and open aneurysmorrhaphy were significantly higher in patients receiving immunosuppressive therapy.

Inflammatory mechanisms are known to play an important role in the pathogenesis of AAA.^8,9)^ Moreover, immunosuppressive therapy has been suggested to influence aneurysm sac behavior. Several studies have reported an association between immunosuppressive therapy and accelerated AAA expansion.^10,11)^ In contrast, a recent study by Thanigaimani et al.^12)^ found no significant difference in aneurysm growth between patients receiving immunosuppressive therapy and those who were not. Thus, although the effects of immunosuppressive therapy on AAA sac behavior warrant attention, they remain ill-understood.

In general, decompression of the aneurysm sac occurs after EVAR and is associated with subsequent shrinkage or stabilization of its diameter. Several factors have been reported to be associated with late sac expansion after EVAR, such as persistent endoleaks, endotension, and use of the device outside the instructions for use.^13)^

To our knowledge, the effects of immunosuppressive therapy on aneurysm sac behavior after EVAR have not been well studied. In the present study, the incidence of sac shrinkage was similar regardless of immunosuppressive therapy status, whereas the incidence of sac expansion was significantly higher in patients receiving immunosuppressive therapy. These findings suggest that immunosuppressive therapy may adversely affect sac stabilization after EVAR in certain patients with AAA. In other words, postoperative inflammation may play some role in sac stabilization in certain patients, rather than uniformly across all cases. Although the association did not reach statistical significance, the observation that persistent or late-onset type II endoleaks were present in 7 of 8 patients with late sac expansion suggests that these endoleaks may promote late sac expansion among patients receiving immunosuppressive therapy.

Long-term survival was similar regardless of immunosuppressive therapy, and no aneurysm-related deaths were observed in patients receiving immunosuppressive therapy. These findings suggest that, despite higher rates of early postoperative complications and late sac expansion, EVAR can achieve its primary therapeutic goal of preventing aneurysm-related death even in patients receiving immunosuppressive therapy.

Our results indicate the need for careful and long-term postoperative surveillance after EVAR in patients receiving immunosuppressive therapy, particularly when residual endoleaks are present. Furthermore, they suggest a potential benefit of implementing prophylactic embolization of branching vessels during the initial procedure in this high-risk population.

The present study has several limitations, including its retrospective design and small sample size. Because of the limited number of cases, propensity score matching could not be performed. The increased risk of early postoperative complications and late sac expansion may have been associated not with immunosuppressive therapy itself, but rather with the patients' underlying comorbidities. An additional limitation is the lack of histological or pathological analysis. Nevertheless, we believe that the findings of this study have the potential to contribute to future research on the association between immunosuppressive therapy and AAAs.

## Conclusion

Immunosuppressive therapy was associated with a significant increase in early postoperative complications in patients undergoing EVAR for AAA. Our findings also suggest that immunosuppressive therapy may adversely affect sac stabilization after EVAR in certain patients.
